# Treatment of childhood osteoporosis – current and future perspectives

**DOI:** 10.1186/1687-9856-2015-S1-O11

**Published:** 2015-04-28

**Authors:** Craig Munns

**Affiliations:** 1 The Children’s Hospital at Westmead, Sydney, NSW, Australia

## 

Bisphosphonates are the mainstay of medical therapy in the fracturing child with osteoporosis. The majority of the data in children pertains to intravenous pamidronate use in children and adolescents with osteogenesis imperfecta (OI), where pamidronate has been associated with improvements in bone mineral density, cortical thickness, vertebral shape, pain, mobility and height [1]. Side-effects of pamidronate including acute phase response to the initial dose and retardation of bone healing have also become apparent. To date, there have been no reports of osteonecrosis of the jaw. The best functional outcomes occur when bisphosphonates are given as part of a multidisciplinary approach to treatment.

More recently, bisphosphonates have been used to treat other primary and secondary osteoporotic disorders e.g. immobility and glucocorticoid. Zoledronate is a third generation bisphosphonate with a potency 100-200 times that of pamidronate. Even though both pamidronate and zoledronate have a similar mechanism of action, zoledronate has potential advantages over pamidronate in the management of paediatric bone disorders due to its shorter infusion time and longer duration of action. Zoledronate has been shown to be effective in the management of osteogenesis imperfecta [2] and secondary osteoporosis [3]. The optimal regimen for intravenous bisphosphonate use in both the acute and maintenance phase of treatment remains to be developed.

Oral bisphosphonates do not appear to be as beneficial as intravenous bisphosphonates in children. Although they result in increased bone density, they do not improve bone pain or alter bone histomorphometry [4]. Larger studies await publication. Further the use of bisphosphonates in primary fracture prevention in children is yet to be investigated.

Biological agents hold promise for the future. Denosumab (RANKL inhibitor) use in children has been reported but it would appear unlikely it will be used widely. Anti-sclerostin antibodies and Dickkopf-1 (DKK1), two Wnt pathway inhibitors, however are potential treatments for primary and secondary osteoporosis with their potent effects on periosteal bone formation [5].

In summary, bisphosphonates have improved the life of children with significant bone fragility. Their use in primary fracture prevention and the utility of new agents such as anti-sclerostin antibodies and DKK1 require further investigation.
